# Mapping Quantitative Trait Loci (QTL) for Resistance to Late Blight in Tomato

**DOI:** 10.3390/ijms18071589

**Published:** 2017-07-22

**Authors:** Dilip R. Panthee, Ann Piotrowski, Ragy Ibrahem

**Affiliations:** Department of Horticultural Science, North Carolina State University, Mountain Horticultural Crops Research and Extension Center, 455 Research Drive, Mills River, NC 28759, USA; aepiotro@ncsu.edu (A.P.); ragy_ibrahem@ncsu.edu (R.I.)

**Keywords:** late blight, *Phytophthora infestans*, resistance breeding, *Solanum lycopersicum*, tomato

## Abstract

Late blight caused by *Phytophthora infestans* (Montagne, Bary) is a devastating disease of tomato worldwide. There are three known major genes, *Ph-1*, *Ph-2*, and *Ph-3*, conferring resistance to late blight. In addition to these three genes, it is also believed that there are additional factors or quantitative trait loci (QTL) conferring resistance to late blight. Precise molecular mapping of all those major genes and potential QTL is important in the development of suitable molecular markers and hence, marker-assisted selection (MAS). The objective of the present study was to map the genes and QTL associated with late blight resistance in a tomato population derived from intra-specific crosses. To achieve this objective, a population, derived from the crossings of NC 1CELBR × Fla. 7775, consisting of 250 individuals at F2 and F2-derived families, were evaluated in replicated trials. These were conducted at Mountain Horticultural Crops Reseach & Extension Center (MHCREC) at Mills River, NC, and Mountain Research Staion (MRS) at Waynesville, NC in 2011, 2014, and 2015. There were two major QTL associated with late blight resistance located on chromosomes 9 and 10 with likelihood of odd (LOD) scores of more than 42 and 6, explaining 67% and 14% of the total phenotypic variation, respectively. The major QTLs are probably caused by the *Ph-2* and *Ph-3* genes. Furthermore, there was a minor QTL on chromosomes 12, which has not been reported before. This minor QTL may be novel and may be worth investigating further. Source of resistance to *Ph-2*, *Ph-3*, and this minor QTL traces back to line L3707, or Richter’s Wild Tomato. The combination of major genes and minor QTL may provide a durable resistance to late blight in tomato.

## 1. Introduction

Late blight (LB), caused by the oomycete *Phytophthora infestans*, (Montagne, Bary) is one of the most potentially devastating diseases of tomato in areas with high humidity and cool temperatures and can cause 100% crop loss in unprotected tomato fields or greenhouses. Because of its devastating economic impact, this disease has been the subject of intensified pathological and genetic research since the occurrence of the Irish potato famine in the 1840s. Genetic resistance to LB in tomato has been of interest for many years, and three major resistance genes have been identified in the red-fruited tomato wild species *S. pimpinellifolium*, including *Ph-1*, *Ph-2*, and *Ph-3*, which have been mapped to tomato chromosomes 7, 10, and 9, respectively. *Ph-1* is a single dominant gene providing resistance to race T-0, but was rapidly overcome by new races of the pathogen. *Ph-1* was mapped to the distal end of chromosome 7 using morphological markers [1]. However, no molecular marker associated with this resistance gene has been reported. Other genes *Ph-4*, and *Ph-5-1* and *Ph-5-2* have also been reported originating from LA1033 [2] and PSLP153 [3], respectively. However, *Ph-4* turned out to be a QTL and *Ph-5* is yet to be characterized for its biological role towards late blight control. Currently, *P. infestans* race T-1 predominates, rendering the resistance conferred by *Ph-1* ineffective. The resistance conditioned by *Ph-2*, a single incomplete-dominant gene mapped to the lower end of the long arm of tomato chromosome 10 [4], provides partial resistance to several isolates of race T-1 [1,5]. *Ph-2* slows, but does not stop, the disease progress [4]. Furthermore, *Ph-2* often fails in the presence of more aggressive isolates [6,7]. *Ph-2* has been mapped to an 8.4 cM interval on the long arm of chromosome 10 between restriction fragment length polymorphism (RFLP) markers CP105 and TG233 [4]. A much stronger resistance gene, *Ph-3*, was discovered in *S. pimpinellifolium* accessions L3707 and L3708 (a.k.a. LA 1269 or PI365957) at the Asian Vegetable Research and Development Center (AVRDC) in Taiwan [6]. Currently, this gene is much more useful than *Ph-1* and *Ph-2* and confers incomplete dominant resistance to a wide range of *P. infestans* isolates of tomato, including those that overcome *Ph-1* and *Ph-2*. [8]. *Ph-3* has been mapped to the long arm of chromosome 9 near RFLP marker TG591a [8]. Recently, this gene was fine-mapped in the 0.5 cM genomic region of long arm of chromosome 9 in between Indel_3 and P55 molecular markers [9]. Utilizing the fine-map information, and considering the significance of the gene in late blight resistance, it was further characterized by cloning a 24 kb region, which encodes for CC-NBS-LRR protein [10]. Recently, co-dominant sequence characterized amplified region (SCAR) [11] and cleaved amplified polymorphic sequence (CAPS) markers have been developed for use in marker-assisted breeding for *Ph-3* (M. Mutschler, pers. comm.) [12]. Identification of these markers associated with *Ph-3* has been extremely useful to transfer the *Ph-3* gene into the desirable genetic background by marker-assisted selection (MAS). However, it has been suggested that the full resistance conferred by L3707 and L3708 (the original sources of *Ph-3*) is conferred by more than just one *Ph-3* locus. Further, that *Ph-3* alone in either homozygous or heterozygous conditions would not be highly commercially desirable, as it does not confer strong resistance against aggressive isolates such as US-7 and US-17 [13,14]. The presence of yet undetermined additional hypostatic gene(s) present in L3707 is necessary to provide full resistance. Further, it has been determined that there are new *P. infestans* isolates which have overcome the *Ph-3* resistance [8]. However, it appears that a combination of *Ph-2* and *Ph-3* confers strong resistance to such isolates. Recently, several tomato-breeding programs around the world, including the North Carolina State University, Pennsylvania State University, Cornell University, and AVRDC—The World Vegetable Center, have succeeded in transferring LB resistance genes to fresh-market and/or processing tomato breeding lines or hybrid cultivars, using a combination of phenotypic screening and MAS. For example, most recently, several fresh-market tomato breeding lines (e.g., NC1 CELBR (*Ph-2* + *Ph-3*) and NC2 CELBR (*Ph-2* + *Ph-3*)) and hybrid cultivars Plum Regal (*Ph-3*), Mountain Magic (*Ph-2* + *Ph-3*), Mountain Merit (*Ph-2* + *Ph-3*), and Mountain Rouge, have been released by the North Carolina State University Tomato Breeding Program, USA [15,16,17]. Further, more breeding lines and cultivars are in the pipeline from these, and other, tomato breeding programs. However, the availability of more useful PCR-based markers for *Ph-2* and *Ph-3* will make the selection and breeding for LB resistance in tomato more expedient. The objective of the present study was to map the genes and QTL associated with late blight resistance in a tomato population derived from intra-specific crosses.

## 2. Results

Late blight is one of the most devastating diseases for tomato throughout the world. Combining the available genes conferring resistance to the late blight in tomato lines is desirable to combat this devastating disease throughout the world. A population-derived from NC 1CELBR × Fla. 7775 developed, with an objective of introgressing the resistance derived from NC 1CELBR. There was a significant difference (*p* < 0.05) among F2-derived population of NC10175 tomato lines for late blight resistance evaluated at MHCREC at Mills River and MRS at Waynesville, NC over the years. Late blight infestation ranged from 0 to 5, where 0 = no late blight infestation, whereas 5 = plant covered with late blight infestation or dead in 2011 (Table 1). In 2014, the minimum disease was 0.8 and maximum was 5.0, with an average of 2.5. However, minimum and average disease scores were 1 and 3.0 at Mills River, in contrast to 0 and 1.9 at Waynesville, respectively. Similarly, the average disease score in 2015 was 1.1, ranging from 0 to 3.0 (Table 1), probably because there was less late blight pressure in 2015. The average of all experiments scored was 1.9, with a range from 0.3 to 4.0 (Table 1). Although we started with 250 lines in 2011, by the time the study was completed in 2015, there were only 175 lines. In several lines, it was not possible to collect seeds because of disease severity. There was a high level of disease pressure in some lines, whereas others were completely healthy.

**Figure 1:**
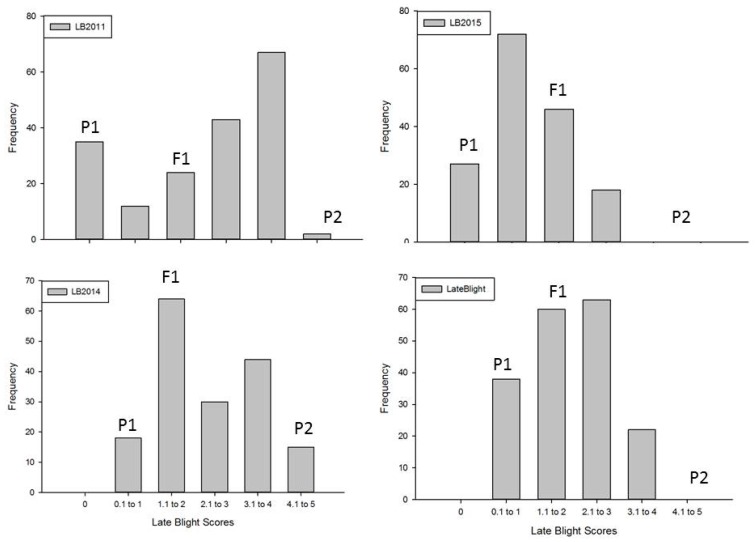
Frequency distribution of late blight resistance in NC10175 population evaluated at Mountain Horticultural Research & Extension Center (MHCREC) at Mills River, and Mountain Research Station (MRS) at Waynesville, North Carolina in 2011, 2014, and 2015. The figure shows the late blight distribution pattern in individual experiment, as well as in the summary data (Late Blight) from each year. In the figure, P1 = NC 1CELBR, P2 = Fla. 7775, and F1 = NC10175.

Late blight resistance comes from NC 1CELBR, and Fla. 7775 is susceptible to the late blight (see Materials and Methods for details). A qualitative resistance is expected to follow the discrete distribution pattern, whereas quantitative resistance is expected to follow a continuous distribution pattern. Late blight distribution patterns in F2, F3 and F4 populations are given in Figure 1. Late blight was normally distributed in an F2, F3, F4 generations and for overall average late blight scores for the entire population (Figure 1).

Comparing late blight scores between years, there was a positive correlation between 2011 and 2014 (*r* = 0.57, *p <* 0.01), 2011 and 2015 (*r* = 0.35, *p <* 0.01), as well as 2014 and 2015 (*r* = 0.42, *p <* 0.01). Comparing locations will be interesting for the sake of disease development. While the population was only in MRS at Waynesville in 2011, there was a very weak positive correlation between MHCREC and MRS in 2014 (*r* = 0.10, *p >* 0.05), whereas it was intermediate in 2015 (*r* = 0.30, *p <* 0.01) (Table 2).

### Quantitative Trait Loci (QTL) Analysis

Two major genes, *Ph-2* and *Ph-3* conferring resistance to late blight, are contributed from NC 1CELBR [18]. *Ph-2* traces back to the Richter’s Wild Tomato, whereas *Ph-3* comes from L3707. QTL associated with late blight resistance in tomato were detected from chromosome 6, 8, 9, 10, and 12. There was one QTL detected from each of chromosome of 6 and 8 with 2.5 and 2.8, LOD scores, respectively, explaining about 2% to 8% of the total phenotypic variability (*R*^2^-value) (data not shown). QTL on chromosome 6 was contributed from P2 (Fla. 7775), since additive effect was positive, indicating that the level of disease resistance was high in the progenies with the alleles from this parent. QTL detected from chromosome 8 in 2011 and MRS, Waynesville 2015 were also contributed from P2 (Fla. 7775).

**Figure 2:**
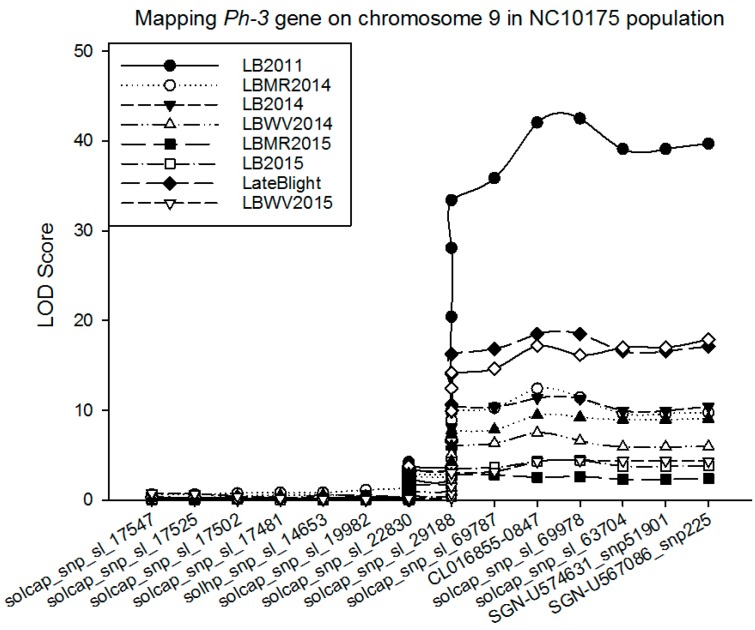
Mapping *Ph-3* on chromosome 9 in segregating tomato population derived from an intra-specific cross. Molecular markers and its position information is given in Table 3. LB2011 = Late blight trial conducted in 2011; LBMR2014 = Late blight trial conducted at MHCREC at Mills River in 2014; LBWV2014 = Late blight trial conducted at MRS, Waynesville, NC in 2014; LB2014 = Average late blight data from MHCREC and MRS in 2014; LBMR2015 = Late blight trial conducted at MHCREC at Mills River in 2015; LBWV2015 = Late blight trial conducted at MRS, Waynesville, NC in 2015; LB2015 = Average late blight data from MHCREC and MRS in 2015. *Y*-axis shows the LOD score and *X*-axis shows the marker labels.

QTL from chromosome 9 were found consistent in all five environments, where *Ph-3*, a major gene conferring resistance to the late blight in tomato, is also located. It was not only consistent, but also a major QTL detected across the environments, with a LOD score of more than 42 explaining 67% of the total phenotypic variation. This QTL is located on about 67 cM position of the chromosome 9 from the telomere where molecular markers CL016855-0847, solcap_snp_sl_69978, and solcap_snp_sl_63704 are located (Table 3; Figure 2). Apparently, this was a major QTL associated with late blight resistance in tomato. Additive effect of the QTL was found to have −1.96, indicating that an individual allele contributed from the resistance parent (P1; NC 1CELBR), and that much disease resistance would increase.

**Figure 3:**
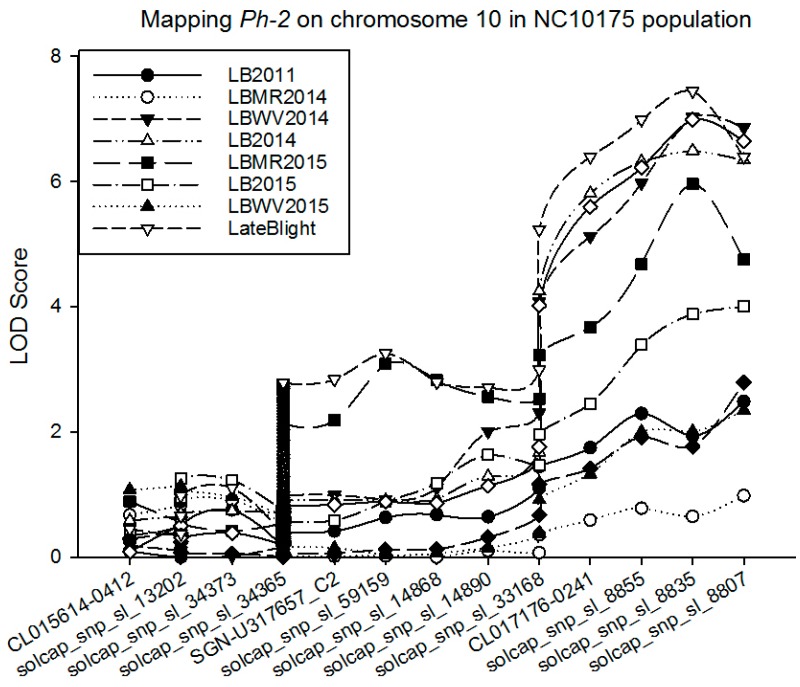
Mapping *Ph-2* on chromosome 10 in segregating tomato population derived from an intra-specific cross. Molecular markers and its position information is given in Table 3. LB2011 = Late blight trial conducted in 2011; LBMR2014 = Late blight trial conducted at MHCREC at Mills River in 2014; LBWV2014 = Late blight trial conducted at MRS, Waynesville, NC in 2014; LB2014 = Average late blight data from MHCREC and MRS in 2014; LBMR2015 = Late blight trial conducted at MHCREC at Mills River in 2015; LBWV2015 = Late blight trial conducted at MRS, Waynesville, NC in 2015; LB2015 = Average late blight data from MHCREC and MRS in 2015. *Y*-axis shows the LOD score and *X*-axis shows the marker labels.

Similarly, there was a major QTL associated with late blight resistance located on chromosome 10, with a LOD score of 7.4, explaining almost 14% of the total phenotypic variation, where *Ph-2* is also located. This QTL was found to be located on about 63 cM of the chromosome. Molecular markers around this QTL are CL017176-0241, solcap_snp_sl_8855, solcap_snp_sl_8835, and solcap_snp_sl_8807 (Table 3; Figure 3). The additive effect of this QTL was estimated to be −0.56, indicating that with an individual allele, 0.56 disease resistance level would increase. Additive resistance alleles were contributed from P1 (NC 1CELBR) for this QTL.

There were also QTLs detected from chromosome 12 with the LOD scores of 3 and 2.6, respectively. However, the level of phenotypic variation explained by an individual QTL was only about 6%, indicating that these were minor QTL (Table 3). This QTL was found to interact with the QTL from chromosome 9 significantly (*p* < 0.05, Table 4). This may be an undetermined additional hypostatic gene(s)/QTL coming from L3707 or Ritcher’s Wild Tomato. Based on the location of the QTL, there were two QTL from chromosome 12 located at a distance of 0.01 and 67 cM with the LOD scores of 3.13 and 2.12, respectively (Table 3). Resistance alleles were contributed from P1 (NC 1CELBR). The magnitude of disease resistance was about −0.30, indicating that this QTL may reduce the disease severity by 0.30 when scored at the scale of 0 to 5.

## 3. Discussion

The objective of the present study was to map the genes and QTL associated with late blight resistance in a tomato population derived from intra-specific crosses. As presented in the Results section, there were two major QTL associated with the late blight resistance located on chromosomes 9 and 10. These had LOD scores of more than 42 and 6, explaining 67% and 15% of the total phenotypic variation, respectively. This indicated that these are the major genes/QTL from these two chromosomes. A co-dominant gene *Ph-2* from chromosome 10 has been reported conferring resistance to late blight [4]. Similarly, *Ph-3* conferring resistance to LB located on chromosome 9 has been reported [8,20]. Further, *Ph-3* was fine-mapped in an interval of 0.5 cM between two molecular markers Indel_3 and P55, at a distance of 74 kb region [9]. These two molecular markers are from the region of TG591 RFLP marker, which is about 55 cM from the telomere. In the present study, we found the location of the race non-specific (which was found to be US-23 genotype of *P. infestans*) LB resistance QTL from these two chromosomes. It is possible that the gene/QTL from chromosome 9 is *Ph-3*, which is about 12 cM upstream from the fine-mapped location.

*Ph-2* locus conferring partial resistance to late blight was mapped to chromosome 10 in an F2 population derived from *Solanum pimpinellifolium* (West Virginia 700 (WVa700) × HI7996) using amplified fragment length polymorphism (AFLP) molecular markers [4]. It was found to be located on the distal part (60 to 70 cM) of the chromosome, in an interval of 8.4 cM on the long arm of chromosome 10 near molecular markers CP105 and TG233. QTL detected in the present study was also from the same region, in fact, it was very close to TG223.

A race-specific resistance gene *Ph-3* provides resistance to a broader range of isolates of *Phytophthora infestans*. The genotypes with *Ph-1* and *Ph-2* genes were susceptible against multiple isolates of *Phytophthora infestans*, as characterized by Kim and Mutschler [21]. It was believed that there may be an additional minor genetic allele in addition to *Ph-3* to confer complete resistance to LB [21,22]. Chen, et al. [23] mapped the QTL associated with *Ph-3* on chromosome 9 and also mapped an additional QTL from chromosome 2 derived from L3708 (*S. pimpinellifolium*). In the present study, we found the major QTL from chromosome 9 and 10. In addition to that, we also found a minor QTL from chromosomes 6,8 and 12, which may be novel minor QTL. These are important when it was widely believed that there should be additional QTL derived from L3707 and potentially from Richter’s Wild Tomato, which needs to be verified in the future studies. A significant interaction between QTL from chromosomes 9 and 12 indicated that these two QTL may be inter-dependent or QTL from chromosome 12 (minor QTL) may play role in transcription of QTL from chromosome 9, which was found to be the major QTL.

While *Ph-2* and *Ph-3* are single genes conferring resistance to the late blight in tomato, a series of studies have shown to have quantitative resistance involved in tomato originating from wild relatives [24,25,26,27]. The source of resistance traces back to LA716, LA1777, and LA2099 [24,28,29]. In order to make the resistance durable, Li, Liu, Bai, Finkers, Wang, Du, Yang, Xie, Visser and van Heusden [28] have suggested the pyramiding of resistance gene and/or QTL from multiple species. Shandil, et al. [30] demonstrated that the level of resistance can vary in potato even if the resistance gene (R gene) was verified by PCR depending upon the genetic background of the recipient. Their conclusion was to investigate the role of other genes for achieving satisfactory level of resistance in potato using R gene. Jo, et al. [31] discuss about gene stacking approach to achieve the durable resistance in potato. Durable resistance has also been shown by combining the qualitative and quantitative resistance in potato in yet another study [32]. They indicate that there may be nucleotide-binding site–leucine-rich repeat (NBS-LRR) mediated resistance in potato. The role of NBS-LRR has also been shown to cause root knot nematode resistance in pepper [33,34].

Major genes have been identified from chromosome 9 and 10, and while this is consistent with past findings, the uniqueness of the present study is that we found some additional QTL from chromosome 6, 8, and 12 with the same source of resistance as *Ph-2* and *Ph-3*. The presence of undetermined additional hypostatic gene(s)/QTL in L3707 is necessary to provide full resistance (R. Gardner, *personal communication*), and the present study has unraveled that to some extent. The pedigree of the present population traces back to the L3707 (*S. pimpinellifolium*), which is also the donor of *Ph-3* [18]. It should be noted that the same line may be the donor of both the major gene *Ph-3*, as well as minor QTL detected on chromosomes 6, 8, and 12 in the conferring LB resistance in tomato. As mentioned before, Kim and Mutschler [13,21] and Irzhansky and Cohen [35] have reported the presence of additional late blight resistance derived from L3708 and L3707, respectively, and the presence of epistatic gene interaction for late blight resistance. This additional L3707/L3708- derived resistance is yet to be mapped and characterized. Since both lines have also been reported to be the source of *Ph-3* gene, it is worth characterizing.

## 4. Materials and Methods

### 4.1. Plant Materials

Tomato breeding line NC 1CELBR was developed at North Carolina State University (NCSU) by Dr. R. G. Gardner. It is a large-fruited fresh-market tomato breeding line with a determinate growth habit and is resistant to LB conferred by *Ph-2* and *Ph-3* genes [18]. Seeds of the susceptible line Fla. 7775 were kindly provided by Dr. Jay Scott, University of Florida. Despite other similar characterisrtics, contrasting LB reactions in NC 1CELBR and Fla. 7775 provided ideal materials to develop a population for genetic mapping studies. Crosses were made in the fall of 2009 at the Mountain Horticultural Crops Research and Extension Center, NCSU, Mills River, NC. The F_2:3_ families were developed in the spring of 2010 by selfing the F_2_. Subsequently, the F_2:3_ population was developed and used for single nucleotide polymorphism (SNP) marker analysis, QTL mapping, and phenotypic evaluation in the field.

### 4.2. Phenotyping for Disease Resistance in Field in 2011

To evaluate resistance to LB in the field, the experiment was conducted in 2011. Seeds were sown in 72 cell flats (56 × 28 cm^2^) in the first week of May. Transplants, at about 6 weeks, were planted by hand in the field. Transplants were planted 45 cm apart in a planting row, with 150 cm between rows. The soil was a clay-loam and the natural day light photoperiod was about 14/10 h with 25–30 °C high and 14–16 °C low day/night temperatures. In the first week of June 2011, the 183, four-week-old greenhouse grown transplants of the F_2_ and F_1_ hybrid (NC 10175), along with susceptible checks (Fletcher, NC123S and NC 30P) and resistant checks (NC 2CELBR, NC 25P, Plum Regal and Mountain Merit), and parents (NC 1CELBR and Fla. 7775) were planted at the Mountain Research Station, Waynesville, NC. This field site was chosen because *P. infestans* inoculum occurs naturally each year. Parents and the F_1_ were planted as a check to make sure that disease developed well in the susceptible parent, and that the resistant parent was healthy even under high inoculum pressure. In the field, a disease rating was performed on the scale of 0 to 5, where 0 = no disease symptoms on the leaf surface area, 1 = symptoms spread over about 20% of the leaf surface area, 2 = symptoms spread over 21–40% of the leaf surface area, 3 = symptoms spread over about 41–60% of the leaf surface area, 4 = symptoms spread over 61–80% of the leaf surface area, and 5 = symptoms spread over 100% of the leaf surface area. Plants that showed defoliation ≤40% were considered as LB resistant and plants exhibited defoliation ≥40% were considered as LB susceptible.

### 4.3. Phenotyping for Disease Resistance in the Field in 2014 and 2015

In 2014 and 2015, the F_2:3_ and F_2:4_ families, parents (NC 1CELBR and Fla. 7775) and control lines were sown into seeding trays in a standard seeding mix (2:2:1 *v*/*v*/*v*) peat moss:pine bark:vermiculite with macro- and micro-nutrients (Van Wingerden International Inc., Mills River, NC, USA). After 10 days, seedlings were transplanted to 72-cell flats (56 × 28 cm^2^). Six plants per genotype were planted with two replications, and the same experiment was conducted in MHCREC, NC.

As the plants were infected with natural inoculum, plants were scored on a scale of 0 to 5, where 0 = no disease symptoms on the leaf surface area, 1 = symptoms spread over about 20% of the leaf surface area, 2 = symptoms spread over 21–40% of the leaf surface area 3 = symptoms spread over about 41–60% of the leaf surface area, 4 = symptoms spread over 61–80% of the leaf surface area, and 5 = symptoms spread over 100% of the leaf surface area. Plants that showed defoliation ≤40% were considered as LB resistant and plants exhibited defoliation ≥40% were considered as LB susceptible.

### 4.4. DNA Isolation and SNP Genotyping

Genomic DNA of young leaf tissues of each line and parent were extracted using DNeasy Plant Mini Kit (Qiagen Inc., Valencia, CA, Spain). A Nano Drop (Model ND-2000, Thermo Scientific Inc., Wilmington, DE, USA) was used to quantify each DNA sample. Approximately 50 ng/µL of DNA was prepared from each sample for SNP genotyping. We used an optimized subset of 384 SNPs markers that were derived from the 7,725 SNP array developed by the Solanaceae Coordinated Agricultural Project (SolCAP) [36,37]. The subset of markers was selected based on polymorphism rates among six fresh market tomato accessions including Fla.7776, Fla.8383, NC33EB-1, 091120-7, Fla. 7775, and NC 1CELBR. In addition, genetic position in the genome based on recombination [36] and physical position were considered as important selection criteria to insure genome coverage. These 384 SNPs were analyzed using the KASP genotyping platform (LGC Genomics, Beverly, MA, USA).

### 4.5. Phenotypic Data Analysis

The disease rating values were calculated from replicated trials to measure resistance to LB in 2011 from the 183 F_2_ plants in the field trial and in 2014 and 2015 from the F_2:3_ and F_2:4_ populations in the field trials. Data from the field experiments were subjected to analysis of variance (ANOVA) using Proc Mixed in SAS 9.3 [38]. Correlation analysis was performed between different environments using PROC CORR procedure of SAS.

### 4.6. QTL Analysis

Out of the 384 SNP markers, only 184 were polymorphic between the two parental lines. NC 1CELBR and Fla. 7775, were used for QTL analysis and to develop genetic maps [39]. Recombination frequencies of the map were converted into genetic distance (cM) using the Kosambi mapping function and calculation of genetic distance between two adjacent SNP was performed [40].

QTL analysis for late blight was carried out by Composite Interval Mapping (CIM) by using QTL Cartographer v 2.5 software [41]. A default threshold of 2.5 was used to declare the presence of QTL in all environments. We used 5 cM scanning steps for the detection of QTL. The relative contribution of genetic component was calculated, and described as the proportion of the phenotypic variance explained. QTLs explaining more than 10% of the phenotypic variance were considered as major QTL, and QTL found in at least two environments were considered to be consistent. Data analysis was repeated by using R software to confirm the presence of QTL and their interaction [19].

Any QTL within 5 cM distance on the same chromosome were regarded as a single QTL. The potential biological function of the SNP markers that were associated with each QTL for resistance to LB were inferred using an in silico approach. The SNP marker sequences were blasted against coding regions of *Arabidopsis thaliana*, rice (*Oryza sativa* L.) and tomato (*S. lycopersicum* L.) databases, genes essential for metabolic pathways, and plant defense-related were identified.

## Figures and Tables

**Table 1:** Descriptive statistics for late blight severity in tomato populations evaluated in various environments in North Carolina.

Variables	LB2011	LBMR2014	LBWV2014	LB2014	LBMR2015	LBWV2015	LB2015	Average Late Blight
Mean	2.5	3.0	1.9	2.5	1.8	0.4	1.1	1.9
Minimum	0.0	1.0	0.0	0.8	0.0	0.0	0.0	0.3
Maximum	5.0	5.0	5.0	5.0	5.0	2.0	3.0	4.0
Standard Deviation	1.5	1.0	2.0	1.2	1.3	0.7	0.8	0.9

LB2011 = Late blight trial conducted in 2011, LBMR2014 = Late blight trial conducted at MHCREC at Mills River in 2014, LBWV2014 = Late blight trial conducted at MRS, Waynesville, NC in 2014, LB2014 = Average late blight data from MHCREC and MRS in 2014, LBMR2015 = Late blight trial conducted at MHCREC at Mills River in 2015, LBWV2015 = Late blight trial conducted at MRS, Waynesville, NC in 2015, LB2015 = Average late blight data from MHCREC and MRS in 2015.

**Table 2:** Correlation analysis between various experiments for the evaluation of late blight in NC10175 population in North Carolina.

Variable	LBMR2014	LBWV2014	LB2014	LBMR2015	LBWV2015	LB2015	Average Late Blight
LB2011	0.34	0.50	0.57	0.29	0.34	0.35	0.79
LBMR2014		0.10	0.54	0.21	0.28	0.27	0.46
LBWV2014			0.91	0.28	0.31	0.34	0.75
LB2014				0.35	0.39	0.42	0.83
LBMR2015					0.30	0.94	0.58
LBWV2015						0.64	0.52
LB2015							0.65

LB2011 = Late blight trial conducted in 2011; LBMR2014 = Late blight trial conducted at MHCREC at Mills River in 2014; LBWV2014 = Late blight trial conducted at MRS, Waynesville, NC in 2014; LB2014 = Average late blight data from MHCREC and MRS in 2014; LBMR2015 = Late blight trial conducted at MHCREC at Mills River in 2015; LBWV2015 = Late blight trial conducted at MRS, Waynesville, NC in 2015; LB2015 = Average late blight data from MHCREC and MRS in 2015.

**Table 3:** Molecular markers on different chromosomes with likelihood of odd (LOD) scores associated with Late blight.

Environment	Chromosome	Markers	Position (cM)	LOD Score	Additive Effect	Dominance Effect	*R*^2^-Value
LB2011	9	solcap_snp_sl_22830	0.32	9.18	−0.33	−2.93	0.81
LBMR2014	9	solcap_snp_sl_22830	0.27	2.75	−1.40	1.48	0.69
	9	solcap_snp_sl_29188	0.66	33.36	−1.66	0.52	0.60
	9	solcap_snp_sl_69787	0.66	35.81	−1.68	0.58	0.64
	9	CL016855-0847	0.67	41.99	−1.69	0.58	0.66
	9	solcap_snp_sl_69978	0.67	42.44	−1.72	0.50	0.67
	9	solcap_snp_sl_63704	0.67	39.05	−1.68	0.52	0.63
	9	SGN-U574631_snp51901	0.67	39.05	−1.68	0.52	0.63
	9	SGN-U567086_snp225	0.67	39.65	−1.71	0.53	0.65
LBWV2014	9	solcap_snp_sl_22830	0.40	3.25	−0.59	−0.38	0.19
	9	solcap_snp_sl_29188	0.66	10.01	−0.72	−0.30	0.23
	9	solcap_snp_sl_69787	0.66	10.27	−0.71	−0.30	0.22
	9	CL016855-0847	0.67	12.40	−0.73	−0.42	0.26
	9	solcap_snp_sl_69978	0.67	11.40	−0.71	−0.36	0.24
	9	solcap_snp_sl_63704	0.67	9.62	−0.67	−0.28	0.21
	9	SGN-U574631_snp51901	0.67	9.62	−0.67	−0.28	0.21
	9	SGN-U567086_snp225	0.67	9.74	−0.68	−0.28	0.21
LB2014	9	solcap_snp_sl_29188	0.66	6.09	−1.00	0.32	0.14
	9	solcap_snp_sl_69787	0.66	6.31	−0.98	0.35	0.13
	9	CL016855-0847	0.67	7.49	−1.06	0.33	0.16
	9	solcap_snp_sl_69978	0.67	6.59	−1.02	0.22	0.14
	9	solcap_snp_sl_63704	0.67	5.92	−0.94	0.36	0.13
	9	SGN-U574631_snp51901	0.67	5.92	−0.94	0.36	0.13
	9	SGN-U567086_snp225	0.67	5.96	−0.95	0.36	0.13
LBMR2015	9	solcap_snp_sl_22830	0.61	3.30	−0.42	−0.24	0.07
	9	solcap_snp_sl_29188	0.66	10.44	−0.81	−0.30	0.21
	9	solcap_snp_sl_69787	0.66	10.37	−0.78	−0.25	0.20
	9	CL016855-0847	0.67	11.36	−0.80	−0.24	0.22
	9	solcap_snp_sl_69978	0.67	11.27	−0.78	−0.30	0.21
	9	solcap_snp_sl_63704	0.67	9.95	−0.75	−0.23	0.19
	9	SGN-U574631_snp51901	0.67	9.95	−0.75	−0.23	0.19
	9	SGN-U567086_snp225	0.67	10.41	−0.78	−0.26	0.21
LBWV2015	9	solcap_snp_sl_22830	0.61	2.91	−0.21	−0.13	0.07
	9	solcap_snp_sl_29188	0.66	7.74	−0.37	0.07	0.17
	9	CL016855-0847	0.67	9.46	−0.38	0.12	0.19
	9	solcap_snp_sl_69978	0.67	9.22	−0.38	0.08	0.19
	9	solcap_snp_sl_63704	0.67	8.97	−0.37	0.09	0.18
	9	SGN-U574631_snp51901	0.67	8.97	−0.37	0.09	0.18
	9	SGN-U567086_snp225	0.67	9.05	−0.38	0.09	0.19
Late Blight	9	solcap_snp_sl_29188	0.66	3.46	−0.39	0.07	0.09
	9	solcap_snp_sl_69787	0.66	3.66	−0.38	0.09	0.09
	9	CL016855-0847	0.67	4.31	−0.42	0.05	0.10
	9	solcap_snp_sl_69978	0.67	4.44	−0.43	0.02	0.11
	9	solcap_snp_sl_63704	0.67	3.77	−0.39	0.07	0.09
	9	SGN-U574631_snp51901	0.67	3.77	−0.39	0.07	0.09
	9	SGN-U567086_snp225	0.67	3.80	−0.40	0.08	0.09
LBMR2014	10	solcap_snp_sl_8807	0.64	2.48	−0.34	−0.03	0.02
LB2014	10	solcap_snp_sl_14890	0.60	2.01	−0.58	0.24	0.04
	10	solcap_snp_sl_33168	0.62	4.07	−0.86	0.28	0.09
	10	CL017176-0241	0.63	5.12	−0.95	0.23	0.11
	10	solcap_snp_sl_8835	0.63	7.01	−1.08	0.52	0.14
	10	solcap_snp_sl_8807	0.64	6.85	−1.10	0.41	0.14
LBMR2015	10	solcap_snp_sl_33168	0.62	4.24	−0.49	−0.08	0.08
	10	CL017176-0241	0.63	5.80	−0.55	−0.08	0.09
	10	solcap_snp_sl_8835	0.63	6.48	−0.59	−0.01	0.10
	10	solcap_snp_sl_8807	0.64	6.33	−0.61	0.03	0.10
	10	solcap_snp_sl_33168	0.62	4.01	−0.28	−0.02	0.09
	10	CL017176-0241	0.63	5.59	−0.31	−0.04	0.11
	10	solcap_snp_sl_8835	0.63	6.98	−0.36	0.05	0.14
	10	solcap_snp_sl_8807	0.64	6.64	−0.36	0.02	0.13
LB2015	10	solcap_snp_sl_34365	0.54	2.00	−0.49	0.19	0.07
	10	SGN-U317657_C2	0.60	2.18	−0.47	0.18	0.06
	10	solcap_snp_sl_59159	0.60	3.08	−0.54	0.36	0.09
	10	solcap_snp_sl_33168	0.62	3.22	−0.61	0.06	0.10
	10	CL017176-0241	0.63	3.67	−0.65	−0.03	0.10
	10	solcap_snp_sl_8835	0.63	5.96	−0.79	−0.20	0.16
	10	solcap_snp_sl_8807	0.64	4.74	−0.74	0.02	0.13
Late Blight	10	CL017176-0241	0.63	2.45	−0.33	−0.01	0.06
	10	solcap_snp_sl_8835	0.63	3.88	−0.42	0.02	0.09
	10	solcap_snp_sl_8807	0.64	4.00	−0.44	0.06	0.10
LBWV2014	12	solcap_snp_sl_12664	0.00	2.21	−0.30	0.10	0.04
	12	solcap_snp_sl_1490	0.01	3.10	−0.37	0.07	0.06
	12	solcap_snp_sl_1504	0.01	3.13	−0.37	0.07	0.06
	12	CL015195-0336	0.01	3.13	−0.37	0.08	0.06
	12	solcap_snp_sl_1525	0.01	2.91	−0.34	0.11	0.06
LB2014	12	solcap_snp_sl_14415	0.60	1.86	−0.27	0.62	0.04
LBMR2015	12	solcap_snp_sl_1525	0.01	2.16	−0.32	0.01	0.03
Late Blight	12	solcap_snp_sl_14415	0.60	2.58	−0.06	0.41	0.06

LB2011 = Late blight trial conducted in 2011; LBMR2014 = Late blight trial conducted at MHCREC at Mills River in 2014; LBWV2014 = Late blight trial conducted at MRS, Waynesville, NC in 2014; LB2014 = Average late blight data from MHCREC and MRS in 2014; LBMR2015 = Late blight trial conducted at MHCREC at Mills River in 2015; LBWV2015 = Late blight trial conducted at MRS, Waynesville, North Carolina in 2015;. LB2015 = Average late blight data from MHCREC and MRS in 2015. Consistent quantitative trait loci (QTL) are highlighted in the table.

**Table 4:** Evaluation of epistatic effect of quantitative trait loci (QTL) associated with late blight resistance from chromosome 9 and 12 in tomato. Table presents the direct outputs including model components and parameter estimates from the R-software analysis [19]. Model formula Y ~ Q1 + Q2.

Source	Degree of Freedom	Sum of Square	Mean Sum of Square	Likelihood of Odd (LOD)	%var	*p*-Value (χ2)	*p*-Value (F)
Model	4	28.03788	7.009469	2.790598	7.080374	0.012026	0.013657
Error	170	367.9564	2.164449				
Total	174	395.9943					
Estimated effects:							
Parameters	Estimates	Standard Error	*t*-Value				
Intercept	2.50944	0.11355	22.101				
9@48.0a	0.68239	0.20929	3.261				
9@48.0d	−0.29844	0.37945	−0.787				
12@12.3a	0.01011	0.16344	0.062				
12@12.3d	0.16303	0.22532	0.724				
